# Cellular Metabolic Network Analysis: Discovering Important Reactions in *Treponema pallidum*

**DOI:** 10.1155/2015/328568

**Published:** 2015-10-01

**Authors:** Xueying Chen, Min Zhao, Hong Qu

**Affiliations:** Center for Bioinformatics, State Key Laboratory of Protein and Plant Gene Research, College of Life Sciences, Peking University, Beijing 100871, China

## Abstract

*T. pallidum*, the syphilis-causing pathogen, performs very differently in metabolism compared with other bacterial pathogens. The desire for safe and effective vaccine of syphilis requests identification of important steps in *T. pallidum*'s metabolism. Here, we apply Flux Balance Analysis to represent the reactions quantitatively. Thus, it is possible to cluster all reactions in *T. pallidum*. By calculating minimal cut sets and analyzing topological structure for the metabolic network of *T. pallidum*, critical reactions are identified. As a comparison, we also apply the analytical approaches to the metabolic network of *H. pylori* to find coregulated drug targets and unique drug targets for different microorganisms. Based on the clustering results, all reactions are further classified into various roles. Therefore, the general picture of their metabolic network is obtained and two types of reactions, both of which are involved in nucleic acid metabolism, are found to be essential for *T. pallidum*. It is also discovered that both hubs of reactions and the isolated reactions in purine and pyrimidine metabolisms play important roles in *T. pallidum*. These reactions could be potential drug targets for treating syphilis.

## 1. Introduction

As data for molecular interactions and sequenced genomes in microorganisms are increasing rapidly, network analysis provides a novel perspective for studies on the metabolism of microorganisms. When it comes to human beings, the study of pathogens is highly related with the treatment of and drug development for infectious and inflammatory diseases. Recent studies have revealed important facts about the metabolisms that microorganisms, especially pathogens, have developed mechanisms to escape from the prevention process of the elaborate network of the human immune system [1, 2]. In this paper,* T. pallidum*, the causative agent of syphilis, is analyzed as a successful demonstration.

*T. pallidum* is a spirochete as well as a phylogenetically ancient and distinct bacterial group. Despite its discovery almost a century ago,* T. pallidum* continues to be an enigma. The problem confronted by syphilis researchers is their failure in cultivating* T. pallidum* in artificial medium [3]. For years, although many scientists attempted to cultivate syphilis spirochete, only nonpathogenic treponema was found, while the virulent treponema still escapes cultivation [3, 4]. It is discovered that the failure in cultivation results from the limited biosynthetic capacity and tolerance for the environmental stress of* T. pallidum* that utilizes glycolysis for energy production. It lacks the tricarboxylic acid cycle and oxidative phosphorylation pathways. In addition, it is unable to synthesize enzyme cofactors, fatty acids, and most amino acids [5]. Further research about* Treponema denticola* (*T. denticola*) and* T. pallidum* reveals that many genes and metabolic capabilities present in* T. denticola*, which enable this bacterium to replicate* in vitro*, are absent in* T. pallidum* rendering the latter incapable of sustained replication under similar conditions. Due to these frustrating facts, it is aspiring to perform an analysis of* T. pallidum*'s metabolic network* in silico*.

Systems biology, which provides us with an efficient way to exploring the biological mechanisms, would facilitate the study of* T. pallidum*. Recently, many studies have found numerous important properties of the metabolic networks such as the scale-free topology [6], network robustness [7], and a hierarchy of modules [8]. However, the limitation of computing capability restrains further exploration on the level of the whole organism. It results in attempts to find subnetworks or to cluster the network nodes. Questions about the differences and similarities among subnetworks and how the behaviors change when subnetworks are separated and grouped together are also raised. In spite of topological and graph analysis, many network models and analysis tools have been developed: biochemical reaction network and statistical influence models network [9–14] and constraint-based reconstruction and analysis [15], among which Flux Balance Analysis has been applied to many theoretical analyses [16] because in general all reactions in a system are maintaining a steady state flux. It not only gives the solution space but also provides a quantifiable method to examine various vast reactions.

It is promising to identify the virulence determinants in* T. pallidum* by cellular metabolic network analysis. Previous work only identifies a small number of virulent determinant genes based on the biological functions related to pathogen-host interaction [5]. Interestingly, nearly all of the* T. pallidum* metabolic enzymes genes are not annotated to be virulent factors because the traditional angle for virulent genes is more focused on housekeeping and pathogen-interaction processes. However, accumulated evidences link the metabolic enzymes as virulence factors in the pathogens [17, 18]. Thus reannotation of* T. pallidum* metabolome by comparative genomics strategy may provide further insight into the metabolic virulent factor [3, 5, 19].

Syphilitic gastritis is the case of chronic active gastritis which involves* T. pallidum* and* H. pylori* together [20]. In clinical practice, it is also necessary to demonstrate* T. pallidum* in gastric lesion to confirm the diagnosis [21]. So, in order to further confirm our analysis, we also investigate* H. pylori* as a comparison to find out differences between organisms, especially the factors which are important to their artificial cultivation.

In this paper, we cluster all reactions in* T. pallidum* and group them into different types based on both the Flux Balance Analysis and topological analysis. By computing the stoichiometry matrix's null space, reactions are represented quantitatively and further classified. In addition, critical reactions are identified through minimal cut set calculation. Analysis of* H. pylori* follows as a comparison. This paper closes with a discussion.

## 2. Results 

### 2.1. Clusters of Reactions and the Role Type of Reactions in* T. pallidum*

**Figure 1:**
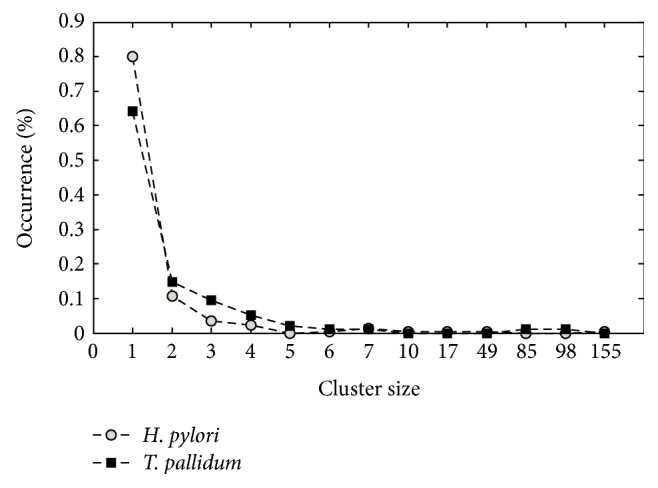
Structure of clusters of* T. pallidum* and* H. pylori*. *x*-axis represents the number of clusters that contain the percentage (indicated by the value on *y*-axis) of reactions.

According to the hierarchical clustering result of 341 reactions in* T. pallidum* obtained from KEGG database, the network has a “hub-structure” (shell-type ordering): there is one or two clusters consisting of a large number of reactions (more than 85 reactions) while the other clusters have only a few reactions (less than 8 reactions) (Figure 1). This type of structure is associated with robust operation and efficient communication in microorganisms.

**Figure 2:**
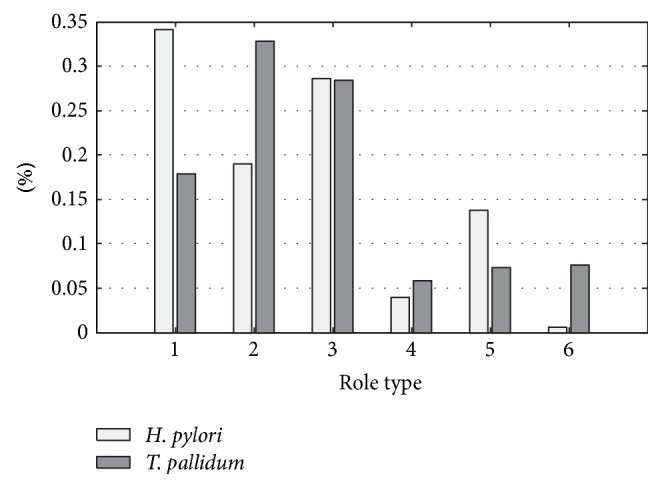
The distribution of role type of the reactions for* T. pallidum* and* H. pylori*. *x*-axis indicates the type of reactions; and *y*-axis indicates the percentage of reactions in the corresponding type.

The role type distributions in Figure 2 imply that most reactions fall into the first three types. According to the definition, reactions in the first three types are closer to their cluster center but far away from the other clusters. This phenomenon indicates a separate functional mechanism in the metabolism. There are 61, 112, 97, 20, 6, and 26 reactions in type 1, type 2, type 3, type 4, type 5, and type 6, respectively. Therefore, reactions of type 2 take the most part in all reactions; reactions of type 4 take the least part. Also, the first three types are much more than the last three types. It could be seen from the results that there are many isolated reactions; most reactions are more connected within their own clusters but have a few reactions linking them to other clusters.

### 2.2. Calculation of Minimal Cut Sets for* T. pallidum*

**Figure 3:**
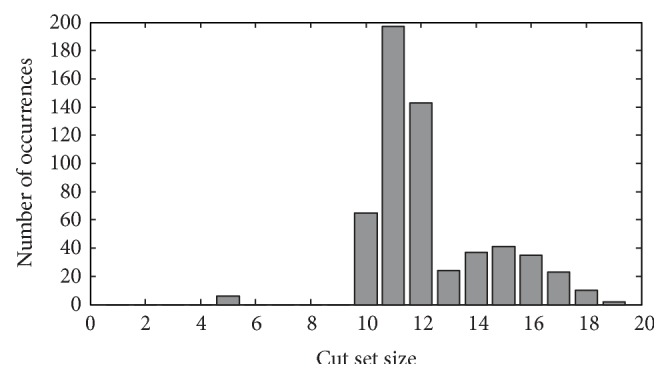
The distribution of the cut set size. *x*-axis indicates the number of reactions in one minimal cut set; and *y*-axis indicates the number of minimal cut sets that have that amount (indicated by *x*-axis) of reactions.

It is known that reactions in nucleotide, purine, and pyrimidine metabolisms play an important role in* T. pallidum*. Here, reactions that take part in minimal cut sets are considered to be crucial to the metabolism [22]. According to our results, 28 reactions that compose 582 minimal cut sets in combination stand out. The overall average cut set size is 11.3127 and the cut set size histogram is shown in Figure 3. These 28 reactions spread in 19 clusters and all of them are type 1 or type 6 reactions except 3 type 2 reactions and 2 type 4 reactions. It means the hutches of reactions and isolated reactions have larger impact on the pathways.

**Figure 4:**
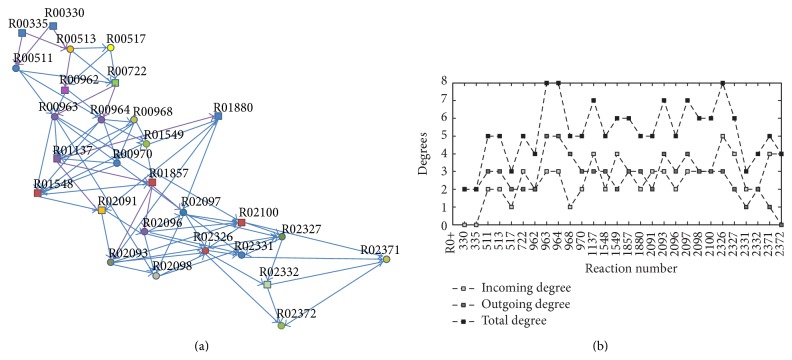
The paths and degrees of reactions in minimal cut sets. (a) Rectangle nodes indicate reactions in purine metabolism; circular nodes indicate reactions in pyrimidine metabolism. The directed edges show the distinct pathways in the minimal cut sets which cannot be reduced further towards the objective reaction. (b) *x*-axis indicates the reactions' ID in KEGG; *y*-axis indicates the degrees of reactions.

The degree distribution for the subnetwork shown in Figure 4 exhibits the combination possibilities for each reaction. All reactions are essential in purine and pyrimidine metabolisms. These combinations further make a distinction among these reactions in minimal cut sets by their significance. For example, we notice the fact that only two upstream reactions (R00300 (D-glucose: NAD+ 1-oxidoreductase) and R00335 (GTP phosphohydrolase)) and one downstream reaction (R02372 (dUTP: cytidine 5′-phosphotransferase)) distinguish themselves since they participate in most minimal cut sets.

### 2.3. Comparison with* H. pylori*

To further explore the functions that the reactions of each role type perform, another microorganism,* H. pylori*, is analyzed.

As a basic comparison, there is a clear similarity between cluster structures of* T. pallidum* and* H. pylori* (see Figure 2), one huge cluster and many small and isolated clusters. This nonuniform distribution meets the recent findings about the scale-free property of cellular networks. It is also related to the highly heterogeneous centrality distribution [23]. Moreover, the similarity of the role type distribution for those two microorganisms (Figure 1) tells that in both organisms' reactions in the same cluster are more likely to be involved with the same metabolites. Thus, the large clusters represent metabolites that have high degrees in the metabolic networks or participate in a lot of reactions and a few nodes with a great number of links, which are often called hubs, hold other nodes together.

**Figure 5:**
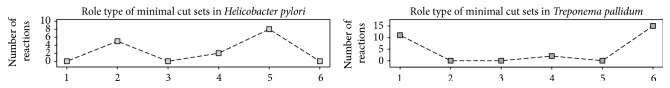
Role type of minimal cut sets in two species. *x*-axis indicates the role type; and *y*-axis indicates the number of reactions in minimal cut sets of the corresponding role type.

In addition, we investigated the role type of reactions that participate in the minimal cut sets and found out that type 4 reactions occur in both microorganisms. However, type 1 and type 6 only exist in* H. pylori* while type 2 and type 5 only exist in* T. pallidum* (Figure 5). The complementarity appears in all role types except types 3 and 4. It leads to the conclusion that if* H. pylori* contaminates the medium where* T. pallidum* grows, biocide that disturbs type 2 and type 5 reactions would be a nice choice since it helps to kill* H. pylori* without affecting* T. pallidum*. In contrast, if* T. pallidum* is the target, impairment of type 1 and type 6 reactions could be a goal to achieve. However, type 4 reactions are essential for both microorganisms which may be untouched in purification but could be a potential target for efficient broad spectrum antibiotic.

## 3. Discussion 

The structure of cellular networks underlying the cellular functions and regulation appeals to researchers to reveal their relationship. Here, we analyze all metabolic reactions in one microorganism by both topological and quantitative methods to discover critical reactions. Although further experimental and clinical verification is still needed, computer simulation and analysis, along with traditional bioinformatics approaches, have frequently been proposed to significantly increase the efficiency of metabolism study of microorganism.

It is worth noticing that all the 28 reactions which are essential to* T. pallidum* are in nucleotide metabolism: 11 (rectangle nodes) take part in purine metabolism and the others (circular nodes) participate in pyrimidine metabolism (Figure 4(a)). This discovery is in concert with the previous work [5, 24]. As mentioned above,* T. pallidum* lacks enzymes that are responsible for the synthesis of fatty acids, amino acids, and so forth. Baseman et al. and the genome analysis both confirmed that* T. pallidum* did not take up the tritiated thymidine and lacked a thymidine kinase (EC 2.7.1.21) but synthesized DNA from uridine nucleotides [5, 24]. Both uridine kinase and uridylate kinase (the gene IDs in KEGG are TP0667 and TP0099, resp.) are present as well as thymidylate kinase (the gene id in KEGG is TP0354).* T. pallidum* possesses the necessary enzymes to synthesize DNA. Among these genes, TP0667 and TP0354 are responsible for 16 reactions (R00513, R00517, R00962, R00964, R00968, R00970, R01548, R01549, R01880, R02096, R02097, R02098, R02327, R02332, R02371, and R02372) in the minimal cut sets, giving strong evidence that both genes and reactions found here are critical in the metabolism of* T. pallidum*.

The purine and pyrimidine metabolism are indispensable constituents critical for synthesis of DNA and RNA and relevant metabolic regulation to all organisms. The mutations of these enzymes often cause lethal effect on the newborn. In total, at least 19 inborn disorders are related to purine and pyrimidine metabolism in human [25]. According to our results from minimal cut set, those reactions related to purine and pyrimidine metabolism tend to be the hub nodes in the entire metabolic network. Previous studies on the biological network revealed that the hubs with large number of interactions are associated with lethality [26]. With the important genetic function, numerous essential enzymes are identified in purine and pyrimidine metabolism. Moreover, many enzymes that catalyze these essential reactions are missed in host human. For example, one of the upstream enzymes glucose 1-dehydrogenase (NAD) not only participates in the majority of minimal cut sets, but also is missed in human. Thus, this enzyme will be one of the best candidates for the follow-up pathogen treatment experimental design.

This method leads us to view the metabolism of microorganism in a new perspective. Topological analysis has been used for drug target selection [27]. Calculated elementary modes of human parasite* Trypanosoma brucei* find out that all three modes obtained are in agreement with experimental observations. Klamt and Gilles have verified the relationship between elementary modes and minimal cut sets [22]. However, interpreting topological calculation, we should keep in mind that this kind of analysis tends to be confined to several determined reactions because the results represent idealized situations. By defining the role type of each reaction, we extend the potential reactions and determine that nucleic acid metabolism is extraordinary important, which could be verified by syphilis pathogenesis. It is reported that virtually every gene in* T. pallidum* is expressed during testicular infection of rabbits [28]. Previous research indicates that the number of DNA recombination and repair genes of* T. pallidum* takes the least part of its DNA sequence. So, once destroyed or interrupted, it is less likely that* T. pallidum* could repair its DNA or RNA to survive [4]. Moreover, Leschine and Canale-Parola [29] discover that treponemal RNA polymerases are resistant to rifampicin but rifampicin is toxic to* H. pylori* [30]. All the results are in agreement with our findings about* T. pallidum* and* H. pylori*.

One of the great current challenges in bioinformatics is to correlate the simple linear world of nucleotide sequence with the nonlinear world of cellular function [31]. In this paper, minimal cut set analysis with FBA provides us with a new combined method. The cell can be approached from top to the bottom, starting from the network's scale-free and hierarchical nature and moving to the organism-specific molecules. By clustering the reactions, we characterize each reaction and determine their importance according to the topological calculation of minimal cut sets. On the other hand, time is also an appealing parameter that should be taken into consideration. As the concentrations or fluxes always change, more questions could be answered under the sense. Thus, it is necessary to extend our cellular metabolic analysis in both space and time.

## 4. Conclusions 

The failure in identifying virulent determinant or drug targets of* T. pallidum* results from its “complex” metabolism. The problem is extended to questions about the importance of reactions and the roles of reactions in the metabolism. This paper is concerned with the related questions. The first question is addressed by calculating the minimal cut sets under the frame of whole metabolism. In concordance with the previous biological findings, nucleotide metabolism contains all 28 reactions in minimal cut sets. The question regarding the reaction role is harder since we do not have a universal definition or classification about the reactions. Here, we use a novel way to deal with the difficulty. The reactions are first quantified by Flux Balance Analysis. Then they are clustered through hierarchical clustering analysis. Given the cluster structure of the reactions, different reaction patterns show up with respect to the within-cluster and out-cluster distances. We associate the reactions in minimal cut sets with their role types and thus extend the concept of essential reactions to essential role types. Furthermore, by analyzing* H. pylori* as a comparison, we discover that different types of reactions have various importance in diverse microorganisms.

## 5. Methods

### 5.1. Description of the Dataset

Data used here were obtained from Kyoto Encyclopedia of Genes and Genomes (KEGG) LIGAND database [32]. For each organism, the LIGAND database contains chemical substances, reactions, and enzymes. However, carrier metabolites such as water and ATP were removed manually.

For each organism, only reactions catalyzed by enzymes were considered.

### 5.2. Flux Balance Analysis and Null Space Determination

In most metabolic network analysis, metabolites are treated as nodes while two metabolites are connected if a biochemical reaction exists. However, to explore the connection between reactions directly, we treated each reaction as a node. Reactions are linked by reaction compounds.

Flux Balance Analysis (FBA) [33, 34] provides a way to estimate the flux distribution of reactions in an organism. More importantly, it assigns every reaction a value that is unique to the reaction. In general, the steady state is common for organism; that is, all reactions in the metabolic system are maintaining a steady state flux. So the concentrations of metabolites and reaction rates are constant. Therefore, a principle equation is achieved. Assume that there are *m* metabolites with concentrations (*c*_1_,…, *c*_*m*_), *r* reactions with fluxes (*r*_1_,…, *r*_*r*_), and *N* is the *m∗r* stoichiometry matrix with element *n*_*ij*_ to be coefficient for metabolite *j* in reaction *i*:

(1) $$\begin{array}{c} 0=\frac{dc_{i}\left(t\right)}{dt}≅\overset{r}{\underset{i=1}{\sum }}n_{ij}r_{i}. \end{array}$$

In a matrix notation,

(2) $$\begin{array}{c} 0=N\overset{⃑}{r}, \end{array}$$

where $\overset{⃑}{r}=(r_{1},\ldots ,r_{r})$.

The mass balance equation above represents the principle constraint of FBA. Additional constraints or objectives are added to maximize the growth in terms of glucose, acetate, glycerol, and so forth, with excess energy production in form of ATP. The physicochemical constraints represent a set of linear equations: $C\vec{r}$, where *C* is *r∗*1 defining the weights of fluxes: fluxes that produce glucose, acetate, glycerol, and ATP have weight 1, fluxes that consume glucose, acetate, glycerol, or ATP have weight −1, and the others have weight 0. Combining these constraints, FBA can be presented as a linear programing problem:

(3) max⁡ Cr→s.t. 0=Nr⃑,

where *N* is the stoichiometry matrix and *C* defines the weights of fluxes in the objective function. To solve this linear programming problem, it is noticed that all stationary flux distributions lie in the right null space of stoichiometry matrix. It can be further derived that the null space could be spanned by its kernel which is indicated by *r∗k* matrix *K*, assuming that the rank of kernel is *k*. Thus, the kernel represents the basis of the steady states of an organism [35]. In addition, each row of the kernel is associated with a reaction which is a crucial fact to quantify the reactions. However, there are infinite representations of the kernel because vectors in the null space are linearly correlated. Given this drawback, constraining the kernel to be an orthogonal matrix may be a solution. It could be proved that the orthogonal matrix is unique for each stoichiometry matrix. Denote by ${\vec{b}}_{1},\ldots ,{\vec{b}}_{k}$ the column vectors of the orthogonal kernel. Then any flux in the steady state can be written as a linear combination of the basis; that is, $\vec{r}=c_{1}{\vec{b}}_{1}+\cdots +c_{k}{\vec{b}}_{k}$, where *c*_*i*_'s are coefficients. Among all the fluxes which satisfy the steady state condition, those with the largest growth rate are the desired states. In other words, the boundary surface of the null space depicts the fluxes that achieve the cellular objective. Depending on the collinearity between the weight matrix and the stoichiometry matrix, the boundary surface could be a set of fluxes or a subspace of the null space. In the latter case, the subsets of the orthogonal basis that span the subspace are the fluxes that quantify the reactions.

### 5.3. Hierarchical Clustering of Reactions

As the flux of a reaction is correlated with its reaction rate, the value of the flux could be used to cluster reactions based on their reaction rates. Here, hierarchical clustering is used to group the reactions.

Firstly, find out the similarities between each pair of reactions according to their “distance.” If two reactions are associated with vector *x*_*r*_ = (*x*_*r*1_, *x*_*r*2_,…, *x*_*rk*_) and vector *x*_*s*_ = (*x*_*s*1_, *x*_*s*2_,…, *x*_*sk*_), respectively, the distance is defined bellow:

(4) $$\begin{array}{c} d_{rs}=1-\frac{\left(x_{r}-{\overset{-}{x}}_{r}\right){\left(x_{s}-{\overset{-}{x}}_{s}\right)}^{\prime }}{\sqrt{\left(x_{r}-{\overset{-}{x}}_{r}\right){\left(x_{r}-{\overset{-}{x}}_{r}\right)}^{\prime }}\sqrt{\left(x_{s}-{\overset{-}{x}}_{s}\right){\left(x_{s}-{\overset{-}{x}}_{s}\right)}^{\prime }}}, \end{array}$$

where ${\overset{-}{x}}_{r}=(1/k)\sum_{j}^{}x_{rj}$ and ${\overset{-}{x}}_{s}=(1/k)\sum_{j}^{}x_{sj}$.

Therefore, when the two reactions are negatively correlated, meaning that the correlation between associated vectors is −1, distance *d*_*rs*_ reaches the maximum value of 2; when the two reactions are positively correlated implying that correlation between associated vectors is 1, distance *d*_*rs*_ reaches the minimum value of 0.

Secondly, group the reactions into a hierarchical tree with regard to the distances. The distance between two clusters is defined by the smallest distance between points in the clusters. Suppose there are *n*_*r*_ reactions in cluster *r* and *n*_*s*_ reactions in cluster *s*. Denote by *x*_*i*_^*r*^ the *i*th reaction in cluster *r* and denote by *x*_*j*_^*s*^ the *j*th reaction in cluster *s*. The distance between clusters *r* and *s* is

(5) dr,s=min⁡dist⁡xir,xjs,  i∈1,…,nr,  j∈1,…,ns,

where dist(*x*_*i*_^*r*^, *x*_*j*_^*s*^) is defined above (4).

Then treat a cluster as a new point to group and so on until we eventually get only one cluster and the hierarchical tree of reaction nodes.

Finally, it is reasonable to cut the tree at the middle level when we need to determine where to cut the hierarchy tree into clusters. In fact, it is shown that there is a wide range of cutoff levels leading to the same results.

### 5.4. Minimal Cut Set Calculation

Elementary mode can be defined as the smallest subnetwork enabling the metabolic system to operate in steady state [25]. It enables us to get insight into cell functions. Elementary mode analysis has been used to predict phenotype and gene expression ratio [36]. It is a “forward” way to model and investigate. On the other hand, analyzing the minimal cut sets (MCS) is an opposite perspective [22]. Klamt and Gilles defined a cut set as a set of reactions (with respect to a defined objective reaction) if after the removal of these reactions from the network no feasible balanced flux distribution involves the objective reaction.

So, for the purpose of finding critical reactions to a specific microorganism, we calculated the minimal cut sets in an organism because the removal of all reactions contained in an MCS could result in a dysfunction of the objective reaction from a perspective of the network structure; and removing a complete MCS from the network could also repress certain functioning. However, other pathways might still be active [22]. These properties of minimal cut sets help to find out species-specific reactions that are essential for pathogen but do not affect the hosts, which could be promising drug targets. We use FluxAnalyzer [37] to do the calculation and use Medusa [38] to draw the map of minimal cut sets.

In addition, to identify important reactions, we pay attention to the degree distribution of reactions in this particular subnetwork formed by reactions that compose minimal cut sets. The total degree is defined as the number of links that a node has. In a directed map for a certain node, an incoming degree is the number of links that point to the node while an outgoing degree is the number of links that start from it. Degree distribution would help us to gain insight into the global structure of the subnetwork.

### 5.5. Role Identification

From the perspective of distance, reactions have different connections either within their own clusters or outside their own clusters. As the reactions have been grouped into different clusters, we further classified reactions into six types.

If *r*_*i*_ = (*r*_*i*1_, *r*_*i*2_,…, *r*_*ik*_) is the vector associated with reaction *i* belonging to cluster *c*, *R*_*c*_ is the average of *r*_*i*_ for all *n* reactions in cluster *c*; that is, *R*_*c*_ = (1/*n*)∑_*i*=1_^*n*^ *r*_*i*_ = (*R*_*c*1_,…, *R*_*ck*_). The within-cluster distance of reaction *i* is defined as *d*_*i*_ = ∑_*j*=1_^*k*^(*r*_*ij*_ − *R*_*cj*_)^2^. Moreover, if there is only one reaction in the cluster, the within-cluster distance is defined to be 0, which is in concert with the former definition.

Suppose there are *s* clusters in all and the average for each cluster is *R*_1_,…, *R*_*s*_, respectively. The out-cluster distance for reaction *i* is defined as *D*_*i*_ = ∑_*c*=1_^*s*^∑_*j*=1_^*k*^(*r*_*ij*_ − *R*_*cj*_)^2^.

**Figure 6:**
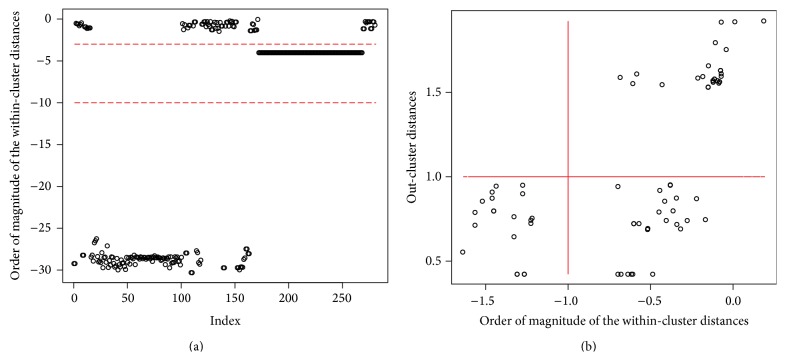
Distributions of within-cluster distances and out-cluster distances for* T. pallidum*. (a) Distribution of the order of magnitude of within-cluster distances. (b) Scatterplot of the order of magnitude of the within-cluster distances against the out-cluster distances for reactions with large within-cluster distances.

Then each reaction is classified according to their scores: *d*_*i*_ and *D*_*i*_. To classify the reactions, assume that reactions of the same type have similar connectivity in the subnetwork. Reactions in the clusters that only contain one reaction are isolated reactions which form a stand-only type. The other reactions are classified by the order of magnitude of the within-cluster distances. As seen in the left panel of Figure 6, the within-cluster distances are well separated and have significant differences in order. Reactions in the clusters which have large within-cluster distances are hubs while reactions in the clusters with small but nonzero within-cluster distances are nonhubs or peripheral nodes. Any typical classification method such as *K*-means would classify the reactions into three groups: one with the order around −30, one with the order around −5, and one with the order above −4. For clusters with within-cluster distances larger than 1*e* − 4 or equivalently with the order larger than −4, the order of within-cluster against the out-cluster distances relationship is shown in the right panel of Figure 6. The plot shows clear pattern of three well-separated groups: one has relatively smaller within-cluster distance and two with the largest within-cluster distances. The two groups with the largest within-cluster distances are further classified by the out-cluster distances: provincial hubs which have larger out-cluster distances and connection hubs which have smaller out-cluster distances. Therefore, all reactions other than isolated reactions are further classified into 5 groups. For convenience purpose, integer thresholds are used in the classification but it is worth noting that the classification is quite robust against the specific numerical thresholds. In summary, if *d*_*i*_ = 0, reaction *i* belongs to type 1; if 1*e* − 10 > *d*_*i*_ > 0, reaction *i* belongs to type 2; if 1*e* − 10 > *d*_*i*_ > 1*e* − 4, reaction *i* belongs to type 3; if 1*e* − 1 > *d*_*i*_ > 1*e* − 4, reaction *i* belongs to type 4; if *d*_*i*_ > 1*e* − 1 and *D*_*i*_ < 1, reaction *i* belongs to type 5; if *d*_*i*_ > 1*e* − 1 and *D*_*i*_ > 1, reaction *i* belongs to type 6.

## Supplementary Material

Below is a list of reaction identifiers of *T. pallidum* and *H. pylori* in the KEGG LIGAND database. Each reaction identifier represents a chemical reaction occurs in the microorganism. For example, R00086 is ATP phosphohydrolase which defined by ATP + H_2_O ⟺ ADP + Orthophosphate.
