# Supplementary material for: Cellular Metabolic Network Analysis: Discovering Important Reactions in Treponema pallidum
Source: Biomed Res Int. 2015 Oct 1;2015:328568. doi: 10.1155/2015/328568 (PMC4606156; doi:10.1155/2015/328568)
Supplement: Supplementary file 1 — Below is a list of reaction identifiers of T. pallidum and H. pylori in the KEGG LIGAND database. Each reaction identifier represents a chemical reaction occurs in the microorganism. For example, R00086 is ATP phosphohydrolase which defined by ATP + H2O ⟺ ADP + Orthophosphate. [file 328568.f1.pdf]

All the reactions of *T. pallidum* and *H. pylori*

T. pallidum	*H. pylori*
R00086	R00004
R00089	R00009
R00104	R00017
R00114	R00036
R00124	R00066
R00127	R00081
R00130	R00084
R00137	R00086
R00156	R00104
R00158	R00124
R00161	R00127
R00174	R00130
R00177	R00131
R00183	R00132
R00190	R00136
R00194	R00137
R00206	R00145
R00214	R00146
R00216	R00156
R00217	R00158
R00230	R00161
R00239	R00177
R00257	R00183
R00260	R00190
R00299	R00194
R00315	R00197
R00330	R00199
R00331	R00220
R00335	R00230
R00355	R00235
R00375	R00236
R00376	R00238
R00377	R00243
R00378	R00245
R00379	R00248
R00382	R00253
R00390	R00256
R00401	R00257
R00431	R00260
R00434	R00261
R00435	R00267
R00437	R00268
R00438	R00275
R00439	R00289
R00440	R00291

R00441	R00299
R00442	R00310
R00443	R00316
R00444	R00321
R00470	R00330
R00471	R00331
R00483	R00332
R00511	R00335
R00512	R00336
R00513	R00351
R00516	R00355
R00517	R00361
R00549	R00375
R00570	R00376
R00571	R00377
R00573	R00378
R00597	R00379
R00600	R00380
R00630	R00382
R00658	R00396
R00660	R00401
R00694	R00408
R00704	R00410
R00722	R00412
R00725	R00415
R00726	R00416
R00734	R00424
R00760	R00425
R00764	R00428
R00768	R00435
R00771	R00437
R00811	R00438
R00835	R00439
R00842	R00440
R00844	R00441
R00857	R00442
R00867	R00443
R00876	R00444
R00896	R00451
R00921	R00470
R00942	R00471
R00943	R00475
R00945	R00480
R00959	R00485
R00962	R00489
R00963	R00490
R00964	R00494

R00966	R00511
R00967	R00549
R00968	R00551
R00970	R00566
R01015	R00568
R01030	R00570
R01049	R00571
R01056	R00573
R01057	R00575
R01061	R00582
R01063	R00586
R01066	R00590
R01067	R00597
R01068	R00600
R01070	R00601
R01074	R00602
R01092	R00625
R01122	R00658
R01126	R00660
R01137	R00674
R01139	R00694
R01140	R00707
R01150	R00708
R01220	R00722
R01227	R00734
R01229	R00742
R01248	R00746
R01251	R00762
R01268	R00768
R01280	R00771
R01321	R00835
R01326	R00842
R01327	R00844
R01330	R00883
R01334	R00888
R01353	R00896
R01401	R00897
R01470	R00921
R01512	R00926
R01518	R00942
R01528	R00943
R01529	R00944
R01547	R00945
R01548	R00963
R01549	R00965
R01560	R00985
R01561	R00986

R01569	R00999
R01600	R01015
R01625	R01036
R01641	R01041
R01654	R01049
R01655	R01056
R01662	R01057
R01663	R01061
R01664	R01063
R01665	R01067
R01724	R01068
R01786	R01070
R01799	R01073
R01801	R01074
R01818	R01078
R01829	R01082
R01830	R01083
R01857	R01090
R01863	R01117
R01876	R01122
R01880	R01126
R01909	R01130
R01961	R01134
R01964	R01135
R01965	R01137
R01968	R01150
R01969	R01177
R02016	R01196
R02017	R01197
R02018	R01199
R02019	R01214
R02024	R01220
R02035	R01226
R02073	R01227
R02088	R01229
R02091	R01231
R02093	R01248
R02094	R01251
R02096	R01253
R02097	R01257
R02098	R01262
R02100	R01288
R02147	R01291
R02237	R01314
R02241	R01316
R02294	R01324
R02295	R01325

R02297	R01341
R02299	R01342
R02301	R01354
R02323	R01374
R02326	R01397
R02327	R01401
R02331	R01403
R02332	R01466
R02342	R01512
R02371	R01513
R02372	R01518
R02433	R01529
R02484	R01547
R02493	R01561
R02556	R01569
R02557	R01600
R02568	R01624
R02619	R01625
R02623	R01626
R02624	R01641
R02630	R01654
R02631	R01655
R02704	R01658
R02719	R01662
R02736	R01664
R02738	R01687
R02739	R01714
R02740	R01728
R02748	R01771
R02760	R01773
R02780	R01775
R02783	R01780
R02788	R01786
R02848	R01797
R02849	R01799
R02850	R01800
R02865	R01801
R02867	R01818
R02868	R01826
R02918	R01827
R02961	R01829
R02971	R01830
R03005	R01845
R03018	R01857
R03020	R01863
R03035	R01867
R03038	R01870

R03076	R01899
R03191	R01900
R03192	R01920
R03193	R01968
R03232	R01969
R03284	R01993
R03291	R02003
R03293	R02016
R03313	R02017
R03319	R02018
R03321	R02019
R03346	R02024
R03530	R02029
R03646	R02035
R03648	R02036
R03650	R02054
R03654	R02055
R03655	R02060
R03656	R02088
R03657	R02090
R03658	R02093
R03659	R02094
R03660	R02098
R03661	R02100
R03662	R02142
R03663	R02147
R03664	R02161
R03665	R02163
R03905	R02166
R03920	R02184
R03940	R02199
R04076	R02237
R04111	R02240
R04112	R02241
R04120	R02272
R04201	R02291
R04206	R02292
R04212	R02294
R04238	R02295
R04241	R02297
R04273	R02323
R04294	R02325
R04295	R02326
R04314	R02331
R04321	R02340
R04361	R02342
R04378	R02412

R04391	R02413
R04393	R02414
R04394	R02433
R04562	R02473
R04572	R02484
R04573	R02508
R04617	R02540
R04771	R02557
R04773	R02568
R04922	R02599
R05027	R02607
R05032	R02619
R05052	R02670
R05132	R02719
R05145	R02722
R05197	R02734
R05199	R02735
R05378	R02736
R05570	R02739
R05577	R02740
R05578	R02748
R05605	R02749
R05628	R02760
R05629	R02767
R05630	R02783
R05633	R02788
R05634	R02792
R05635	R02853
R05636	R02869
R05637	R02918
R05662	R02961
R05688	R02965
R05820	R02971
R05883	R02984
R05884	R03003
R06134	R03005
R06137	R03018
R06172	R03020
R06173	R03035
R06174	R03038
R06212	R03042
R06229	R03051
R06236	R03066
R06237	R03067
R06447	R03083
R06590	R03084
R06613	R03096

R06728	R03132
R06861	R03165
R06863	R03180
R07180	R03182
R07219	R03191
R07246	R03192
R07269	R03193
R07282	R03197
R07283	R03210
R07284	R03223
R07297	R03231
R07389	R03254
R07460	R03260
R07606	R03284
R07671	R03291
	R03293
	R03294
	R03295
	R03321
	R03346
	R03348
	R03350
	R03351
	R03409
	R03411
	R03423
	R03458
	R03459
	R03460
	R03471
	R03503
	R03504
	R03508
	R03509
	R03530
	R03537
	R03538
	R03601
	R03646
	R03650
	R03654
	R03655
	R03656
	R03657
	R03658
	R03659
	R03660

R03661
R03662
R03663
R03664
R03665
R03789
R03867
R03905
R03909
R03916
R03929
R03940
R03970
R03971
R04034
R04109
R04112
R04120
R04144
R04159
R04190
R04198
R04199
R04201
R04206
R04212
R04215
R04238
R04241
R04273
R04294
R04314
R04355
R04361
R04365
R04378
R04385
R04386
R04391
R04429
R04439
R04440
R04444
R04445
R04448
R04475
R04509

R04533
R04534
R04536
R04543
R04559
R04562
R04566
R04567
R04572
R04573
R04606
R04617
R04639
R04657
R04689
R04724
R04726
R04771
R04773
R04780
R04858
R04859
R04935
R04944
R04945
R04946
R04952
R04953
R04955
R04957
R04958
R04960
R04961
R04963
R04964
R04966
R04968
R04969
R04972
R05027
R05028
R05030
R05032
R05033
R05046
R05048
R05051

R05052
R05068
R05069
R05071
R05086
R05135
R05145
R05176
R05197
R05231
R05332
R05378
R05551
R05553
R05577
R05578
R05590
R05605
R05628
R05629
R05630
R05633
R05634
R05635
R05636
R05637
R05662
R05681
R05688
R05692
R05838
R05883
R05884
R06131
R06134
R06137
R06172
R06173
R06174
R06447
R06590
R06613
R06861
R06863
R06895
R07059
R07060

R07166
R07180
R07219
R07246
R07269
R07282
R07297
R07328
R07378
R07460
R07605
R07606
R07607
R07641
R07763
R07860
